# Behavioral healthcare organizations’ experiences related to use of telehealth as a result of the COVID-19 pandemic: an exploratory study

**DOI:** 10.1186/s12913-022-08114-y

**Published:** 2022-06-13

**Authors:** Abby Kisicki, Sara Becker, Michael Chaple, David H. Gustafson, Bryan J. Hartzler, Nora Jacobson, Ann A. Murphy, Stephanie Tapscott, Todd Molfenter

**Affiliations:** 1 Center for Health Enhancement Systems Studies, University of Wisconsin–Madison, 1513 University Ave., WI 53706 Madison, USA; 2 Center for Alcohol and Addictions Studies, Brown University School of Public Health, 121 South Main Street, Providence, RI 02912 USA; 3 Division On Substance Use Disorders, New York State Psychiatric Institute, Columbia University Irving Medical Center, 1051 Riverside Drive, New York, NY 10032 USA; 4 Alcohol & Drug Abuse Institute, University of Washington, 1107 NE 45th Street, Suite 120, Seattle, WA 98105 USA; 5 Institute for Clinical and Translational Research, Community Academic Partnerships Program, University of Wisconsin-Madison School of Nursing, 701 Highland Ave, Madison, WI 53705 USA; 6 Department of Psychiatric Rehabilitation and Counseling Professions, Rutgers, The State University of New Jersey, 675 Hoes Lane West, Piscataway, NJ 08854 USA; 7 Department of Health Policy and Management, Rollins School of Public Health, Emory University, 1518 Clifton Rd., Atlanta, GA 30322 USA

**Keywords:** Telehealth, Virtual services, Mental health, Substance use disorder, Reimbursement, Qualitative research

## Abstract

**Background:**

Due to the COVID-19 pandemic, healthcare providers were forced to shift many services quickly from in-person to virtual, including substance use disorder (SUD) and mental health (MH) treatment services. This led to a sharp increase in telehealth services, with health systems seeing patients virtually at hundreds of times the rate as before the onset of the COVID-19 pandemic.

By analyzing qualitative data about SUD and MH care organizations’ experiences using telehealth, this study aims to elucidate emergent themes related to telehealth use by the front-line behavioral health workforce.

**Methods:**

This study uses qualitative data from large-scale web surveys distributed to SUD and MH organizations between May and August 2020. At the end of these surveys, the following question was posed in free-response form: “*Is there anything else you would like to say about use of telehealth during or after the COVID-19 pandemic?*” Respondents were asked to answer on behalf of their organizations. The 391 responses to this question were analyzed for emergent themes using a conventional approach to content analysis.

**Results:**

Three major themes emerged: COVID-specific experiences with telehealth, general experiences with telehealth, and recommendations to continue telehealth delivery. Convenience, access to new populations, and lack of commute were frequently cited advantages of telehealth, while perceived ineffectiveness of and limited access to technology were frequently cited disadvantages. Also commonly mentioned was the relaxation of reimbursement regulations. Respondents supported continuation of relaxed regulations, increased institutional support, and using a combination of telehealth and in-person care in their practices.

**Conclusions:**

This study advanced our knowledge of how the behavioral health workforce experiences telehealth delivery. Further longitudinal research comparing treatment outcomes of those receiving in-person and virtual services will be necessary to undergird organizations’ financial support, and perhaps also legislative support, for virtual SUD and MH services.

## Background

The coronavirus (COVID-19) pandemic radically transformed substance use disorder (SUD) and mental health (MH) treatment. Due to social distancing requirements, stay-at-home orders, and other precautionary health measures, healthcare providers were forced to quickly shift many services from in-person to virtual, including SUD and MH treatment services [1, 2]. This led to a sharp increase in use of telehealth services, with health systems seeing patients virtually at hundreds of times the rate as before the onset of the COVID-19 pandemic [3]. Medicare, a United States government health insurance program for people 65 and older, which in 2016 lagged in telehealth coverage compared to private insurers and Medicaid, its counterpart for people with low incomes, saw a particularly significant increase in telehealth use: only 0.1% of Medicare primary care visits were delivered virtually before the onset of the COVID-19 pandemic relative to nearly 50% delivered virtually after March 2020 [3, 4]. This spike in telehealth use occurred when SUD and MH treatment were already seeing steady increases in telehealth use. From 2010 to 2017, treatment via telehealth increased rapidly for both SUD and MH disorders [4], but not at the rates of growth experienced following the onset of COVID-19 safety procedures.

Regulatory changes that went into effect at the beginning of the COVID -19 pandemic facilitated the rapid and widespread expansion of telehealth [5]. In the United States, with substantial variability by state, these eased regulations included expanded reimbursement by Medicare, Medicaid, and private insurers; coverage parity or payment parity; provision of telehealth services across state lines; and reimbursement of telephone services at the same rate as in-person face-to-face visits [6]. It is unclear the extent to which these eased regulations will be permanently adopted once the COVID-19 public health emergency comes to an end, leaving providers uncertain in their planning for next steps for telehealth.

Research has shown that many providers indeed hope to continue the expanded use of telehealth after the COVID-19 pandemic. In their 2021 survey of MH care providers around the United States, Guinart et al. [7] reported that 64% of respondents would like to continue using telehealth treatment with at least 25% of their clients and that 53% of these providers would like to continue using telehealth with at least 50% of their clients. Reimbursement for telehealth has often been cited as a barrier by MH [8, 9] and SUD treatment providers [4], even before the COVID-19 pandemic and despite legislative action to address the reimbursement barrier [10]. Although behavioral health treatment organizations have necessarily been integrating telehealth into their practices since the beginning of the pandemic, knowledge of United States behavioral health organizations’ experiences and their plans for the continued use of telehealth after the COVID-19 pandemic remains extremely limited. This study aims to advance knowledge by analyzing qualitative data gathered from web surveys administered in the summer of 2020 [1, 2] about SUD and MH care organizations’ experiences using telehealth. The overarching objective of this analysis was to elucidate emergent themes related to the utilization of telehealth by the front-line behavioral health workforce.

## Methods

This study used data from large-scale web surveys distributed by designated regional Addiction Technology Transfer Centers (ATTCs) and Mental Health Technology Transfer Centers (MHTTCs) to SUD and MH providers, respectively, between May and August 2020 [1, 2]. Respondents were asked to respond on behalf of their organizations. The surveys collected information on how the respondents’ organizations applied virtual services during COVID-19 and their experiences with telehealth use [1, 2]. Results from the original analysis of the surveys showed that the SUD and MH organizations intended to continue delivering many video services and some telephonic services after the COVID-19 pandemic public health safety procedures subside. At the end of these surveys, the following question was posed in free-response form: “*Is there anything else you would like to say about use of telehealth during or after the COVID-19 pandemic?*”.

Out of the 948 SUD and MH survey responses, 391 (41% of) respondents answered this question. These 391 respondents’ self-identified organization setting, organization type, and job function were also pulled from the survey data. Organization settings included rural, small city, suburban, and urban; organization types included specialty treatment, health system, opioid treatment programs, recovery community, Federally Qualified Health Center (FQHC),^1^ and other; job functions included counselor, administrator, physician/prescriber, recovery coach, and other.

One independent coder analyzed the 391 verbatim responses using a conventional approach to content analysis [12], facilitated by NVivo 12 software. As a first step, a research team consisting of three individuals (one psychologist, one qualitative methodologist, one implementation scientist) reviewed all the responses and devised a preliminary coding structure. The independent coder then reviewed the responses and assigned an initial set of preliminary codes. During this first coding pass, the independent coder reviewed codes in an iterative fashion and added more granular themes to the preliminary coding structure as needed. The complete list of themes, along with excerpts from the data, were then reviewed by the research team. In a second pass, the independent coder revisited the data to consolidate repetitive sub-codes and refine and finalize the set of themes.

## Results

### Sample characteristics

Of the 391 respondents, 59.3% were SUD respondents, and 40.7% were MH respondents. The sample consisted of 46.3% counselors, 45.0% administrators, 2.8% physicians/prescribers, 2.6% recovery coaches, and 3.3% other (Table 1). As respondents were answering on behalf of their organization, they were not asked to provide socio-demographic data. Respondents were predominantly in specialty care settings and represented a mix of urban, suburban, and rural areas.

**Table 1:** Sample characteristics

	Frequency	%
**Organization setting**		
Rural	101	25.8
Small city	74	18.9
Suburban	75	19.2
Urban	140	35.8
Blank	1	0.0
**Organization type**		
Specialty treatment	269	68.8
Health system	58	14.8
Opioid treatment programs	25	6.4
Recovery community	18	4.6
FQHC	17	4.4
Other	4	1.0
**Respondent job function**		
Counselor	181	46.3
Administrator	176	45.0
Physician/Prescriber	11	2.8
Recovery coach	10	2.6
Other	13	3.3

### Overview of qualitative emergent themes

Overall, three major themes emerged: COVID-specific experiences with telehealth, general experiences with telehealth, and recommendations to continue telehealth delivery. Each of these themes contained multiple sub-themes, as elaborated in the following sections.

#### COVID-specific telehealth themes

Multiple respondents expressed the view that their organizations would not have begun to use telehealth if not for the onset of the COVID-19 pandemic. Within those responses that specifically commented on the pandemic, three subthemes emerged pertaining to COVID-specific advantages of telehealth adoption: *continued provision of services during the pandemic,* the ability to adhere to *safety and social distancing* guidelines*,* and experiences related to having *children at home*. Another COVID-specific sub-theme was the easing of regulatory restrictions, which facilitated organizations’ adoption of telehealth.

##### Continued provision of services during the pandemic

Many appreciated that virtual services could be used in place of in-person treatment once stay-at-home orders and social distancing requirements began. Respondents conveyed a sense of relief that organizations were able to continue providing services to clients during COVID-19. Some respondents thought that the substitution of telehealth for in-person treatment *“saved lives,” “kept our patients out of the ED and psychiatric hospital,”* and was an *“incredible lifeline”* to clients*.* One respondent stated that increased accessibility over the telephone was beneficial for clients, writing that *“for clients to be able to access help before going into a full-blown crisis is priceless.”* Respondents also expressed gratitude that telehealth allowed them, as individual providers and as a field, to continue performing their jobs. Several comments credited the ability to provide virtual services with having *“saved my job”* and having *“literally saved our organization and likely our clients.”*

##### Safety and social distancing

Respondents reported satisfaction with how telehealth promoted adherence to social distancing guidelines and allowed clients and providers alike to minimize exposure to COVID-19. Telehealth was reported to be particularly beneficial for providers and clients at high personal risk of severe illness from COVID-19.

##### Children at home

To reduce COVID-19 exposure, schools began to conduct learning remotely. As many childcare centers remained closed, this left many parents with the additional challenge of caring for children at home during the day. Respondents appreciated that telehealth allowed employees at their organization (and their clients) to engage with treatment while balancing childcare needs.

##### Easing of regulatory restrictions on telehealth

Many respondents credited the easing of telehealth regulations during COVID-19 with their ability to maintain their use of telehealth. The responses referenced COVID-era policy generally, as well as reimbursement policy specifically. Many referred to the use of telehealth as *“completely dependent”* upon the eased regulations becoming permanent. Some of the respondents expressed uncertainty about how long eased regulations would last. Several noted that the uncertainty “*makes it difficult to do long-term planning/workflow development that includes [telehealth].*”

#### General telehealth themes

The bulk of the verbatim responses shared general experiences with telehealth independent of the COVID-19 pandemic. These ‘general’ responses reflected four subthemes: *organizational conditions affecting ability to adopt telehealth, access, perceived treatment effectiveness, and cost-effectiveness*. Within each of these subthemes, respondents detailed both advantages and disadvantages of using telehealth.

##### Organizational conditions which affect ability to adopt telehealth

A handful of respondents reported positive experiences with the integration of telehealth into their organizational workflow, stating that their organization had made telehealth easy to adopt. Others shared positive feedback about their organization’s access to technology, which allowed them to easily download necessary platforms and navigate telehealth technology, even if they initially experienced a learning curve with setting up virtual services.

By contrast, multiple respondents reported struggles integrating telehealth into their organization’s usual operations. Concerns about confidentiality, security, and encryption on platforms like Zoom were common. Several shared that they received limited support and training from management on adjusting to telehealth, that they were not given the equipment necessary for seamless and proper use of telehealth, and that managing multiple platforms was difficult. This difficulty was in some cases exacerbated by limited staff literacy. One respondent remarked that they struggled to adapt to telehealth at first, prompting them to “*take online courses and webinars to learn [how to use telehealth]”* on their own, as *“[t]he organization [doesn’t] offer any webinars or trainings.”* Two others reported that co-workers were resorting to using HIPAA non-compliant platforms to treat clients due to lack of organizational infrastructure.

##### Access

Multiple respondents remarked on client access to telehealth. Many reported that telehealth removed barriers of access to transportation, distance to treatment, and access to childcare. As one respondent put it, *“No transportation — no problem! Gas is too expensive — no problem! No daycare — no problem!”.*

Several respondents shared their perspective that virtual services opened the door for new populations to access treatment, such as clients with severe anxiety and clients who wish to avoid stigma associated with seeking treatment. Telehealth was also perceived as allowing respondents to provide remote treatment to clients residing in locations distant from them, such as clients in rural areas. As one respondent wrote: “*[T]o have telephonic and telehealth end would mean many clients leaving services. Accessibility is everything, especially for the clients in the upper peninsula that we serve electronically right now. We’d love to see telehealth and telephonic appointment reimbursement continue. We’ve seen such an influx in SUD self-referrals because it is now so much more accessible.”* Some observed that clients kept their telehealth appointments more often than they did with in-person appointments and that clients were generally more engaged in telehealth, with one respondent noting that they *“got better follow through and fewer no-shows.”* In addition, numerous respondents reported that their number of admitted clients increased during telehealth.

While some respondents perceived telehealth as opening up treatment, others perceived telehealth as making treatment less accessible. Several noted that switching to virtual services cut them off from some clients altogether, such as those who have unstable housing, lack adequate privacy for appointments, or refuse to use telehealth. One expressed worry that not enough people in the rural community were aware of telehealth as a treatment option. Additional concerns were that clients were harder to reach, made fewer appointments over telehealth, disengaged from services, or missed more appointments. Respondents also perceived that new clients took longer to engage in virtual services than their preexisting clients. Some respondents reported that their clients missed and rescheduled telehealth appointments more frequently than with in-person appointments. In contrast, others reported that several of their clients had fully disengaged from telehealth.

The most common concern about access to care was clients’ limited access to technology. Many respondents noted that their clients did not have access to the internet, with a few citing poor broadband infrastructure in rural areas. Others reported that their clients did not have cell phones that could support telehealth platforms. Limited cell phone data and limited battery life were cited as problems, as sometimes clients would run out — or fear running out —of their cellular data or battery life if they used virtual services. In addition, respondents perceived some of their clients — such as clients from rural areas, low-income clients, cognitively impaired clients, and older adults — as more prone to having difficulty navigating technology. An overarching worry was that telehealth would exacerbate preexisting barriers to care, especially within historically marginalized client populations and communities of color. Two respondents reported that technology issues raised barriers to continuous access and engagement during sessions, with one respondent adding that this situation posed unanticipated billing issues:

*Cell phone quality & connection problems … can be a problem in meeting minimum session length requirements for billing. For example, I have a client whose phone frequently cuts out every 2 minutes. It gets frustrating for the client & counselor to have to keep calling each other back. It also raises an ethical issue if we’ve only talked for 15 minutes but need to bill for a 25-minute session. If we don’t bill for it, then it’s as if the client wasn’t seen for a session — not fair for the client to be penalized for having a poor-quality phone, or for the program not to get credit for trying to serve the client despite the phone limitations.*

##### Perceived treatment effectiveness

Another key subtheme pertained to the perceived effectiveness of telehealth. Opinions were mixed regarding whether telehealth improved or decreased the effectiveness of the care they were able to offer to clients. As an example, one respondent wrote, “*Truly a mixed bag*. *Works beautifully for some folks and want to continue even after COVID. For others, it is deeply frustrating and they long to come back face-to-face.”*

A handful of respondents reported that clients seemed more open or trusting over telehealth than during in-person sessions, while only respondents whose clients had preexisting trust issues reported lower trust levels. Respondents also appreciated that clients’ engagement in video care from home gave them insight into the client’s home environment.

Several shared their perspective that telehealth was valuable if necessary but could never be an equally effective replacement for in-person treatment. *“It is not an adequate substitute for in-person therapy,”* one respondent wrote, *“but it is better than nothing.”* One of the key trepidations about effectiveness was missing important *“body language cues”* from clients during sessions. For example, one respondent noted, *“without seeing them in person, it is hard to tell [when] they are struggling.”* Several others similarly felt that the *“personal touch”* was missing over telehealth, citing concerns with *“rapport,” “depth,”* and *“connection,”* as well as experiences of *“disconnect.”* One respondent shared that they were missing out on the *“genuine transformation you see when the person comes to the office weekly.”* Others felt that telehealth diminished the rigor of assessment and made it harder to maintain client accountability.

##### Cost-effectiveness

Another subtheme pertained to perspectives of the cost-effectiveness of telehealth. These perspectives were mixed. Some respondents felt that telehealth was more productive and efficient. One respondent commented that *“the more I use it, the more I like it because it saves me time, money, and energy.”* Another said that *“administratively it is cost effective because I do not have staff with as much ‘windshield time’ as they drive across 4 rural counties to provide services.”* Others said that telehealth required a greater investment of work and time, citing technology setup and building rapport with clients.

#### Recommendations to continue telehealth delivery

Three subthemes emerged related to recommendations to advance telehealth: continuing to ease or change telehealth restrictions, investing in broadband infrastructure and institutional support, and combining telehealth and face-to-face intervention.

##### Continued easing of regulations

The most frequent suggestion was to continue telehealth reimbursement and relaxed regulations. *“It would be a shame,”* one respondent said, *“to again implement the limitations.”* Beyond continuation of eased regulations, some respondents took it a step further and advocated for changed regulations. One respondent suggested, *“Let’s change the previous regulations to facilitate the use of telehealth by non-licensed professionals such as recovery coaches, case managers and counselors.”* Another echoed this, saying that *“we need to continue to allow bachelor level and recovery support services staff to use telehealth post-pandemic.”*

##### Investing in broadband infrastructure and institutional support

Another popular suggestion was to invest in expanding access to infrastructure in rural areas to increase access to telehealth services. *“Internet service providers need to MAJORLY step up availability and affordability of internet services in rural areas,*” one respondent said. Meanwhile, another respondent noted that ensuring equitable access to care would require large-scale changes. *“These barriers,”* they said, *“are too large for our non-profit agency to address.”* Institutional support was also viewed as critical. Multiple respondents expressed a desire for more training, technical support, and equipment for staff, as well as increased security and improvement of telehealth platforms.

##### Combining telehealth and in-person care

Finally, several respondents explained that they intended to continue using a combination of telehealth and in-person care after the end of the COVID-19 pandemic. Many shared their perspective that although they do not prefer telehealth to in-person care*,* it should continue as an option for clients. However, it was commonly acknowledged that the ability to offer a hybrid combined model would depend on reimbursement. For example, one respondent wanted their care model to integrate telehealth, but explained that “*without reimbursement ability, it is not an option at all.”*

One respondent took it a step further and argued that telehealth should be viewed as a key part of the future of treatment, explaining: *“I think it’s time to not see telehealth only as an alternative in times of urgent need, but rather as a necessity that requires thorough training, development of sound policies and implementation of secure telehealth systems.”*

## Discussion

This study advances our knowledge of how the behavioral health workforce experiences telehealth delivery. Results revealed three key emergent themes: COVID-specific issues with telehealth, general issues with telehealth, and future directions for telehealth. Across these themes, key issues pertained to access to care, the effectiveness and cost-effectiveness of treatment, and the need for institutional support.

Respondents’ attitudes toward the effectiveness of virtual services were highly consistent with findings of other studies conducted around the same time: A survey administered to nearly 1,500 MH professionals in May and June 2020 found that while respondents endorsed telehealth as important, necessary, and effective, providers gave a relatively lower endorsement of the belief that telehealth is as effective as in-person care [13]. Numerous other studies have established providers’ general preference for in-person care when compared with telehealth [14–16], despite data indicating that in-person care and telehealth have comparable effectiveness [13, 17, 18].

Although multiple hurdles to effective and accessible use of telehealth remain, the responses to this study revealed that telehealth has proven to be an invaluable and convenient tool for SUD and MH treatment organizations. Respondents overwhelmingly praised the ability of telehealth to address the barriers of long distances to treatment and access to transportation. Prior research has cited lack of commute as a key benefit to telehealth, a finding echoed in these results [19, 20]. Telehealth has been noted for its ability to provide access to harder-to-reach populations and was created for this purpose [20]. It is notable that even when the organizations in the current study began to use telehealth for a reason other than initiating deliberate outreach to harder-to-reach demographics, some still observed new clients entering treatment. The reported benefits, many of which were independent of the context of COVID-19, demonstrated that telehealth can provide unique benefits irreproducible by face-to-face treatment and, if used strategically, could potentially act as a tool that enhances and supports the client–clinician relationship [21, 22].

The findings of this study were also highly consistent with elements of Planning and Evaluating Remote Consultation Services (PERCS), a conceptual framework created by Greenhalgh et al. to guide the planning and evaluation of remote consulting services [22]. The PERCS framework consists of eight ‘domains’ — the reason for consulting, the patient, the clinical relationship, the home and family, technologies, staff, the healthcare organization, and the wider system — and focuses attention on the organization’s digital maturity and digital inclusion efforts [22]. Our data aligned well with PERCS’ emphasis on digital maturity and inclusion, as among respondents raising barriers to telehealth, concerns about clients’ access to technology were resoundingly common. The PERCS framework can help organizations prepare solutions to the barriers our data identified. For example, the PERCS framework advocates that organizations should aim for digital inclusiveness by providing support to users who need to acquire digital skills and obtain the technologies necessary to access remote care. Similarly, the PERCS framework advocates for digital maturity by encouraging organizations to ensure the infrastructure needed to circumvent many technology issues cited in this study. Notable in this study was respondents’ frequent citation of the following technology barriers specific to clients’ cellular devices: devices incapable of supporting telehealth software, limited cell phone data or battery life (and clients’ resulting caution not to waste these), and clients not owning a cellular device. Some of the other technology issues cited in this study, such as older adults’ difficulty with technology and limited broadband access in rural or otherwise underserved areas, have been noted as barriers to telehealth and further supported the need for digital inclusion [20]. These barriers introduced a host of ethical issues such as billing for sessions interrupted by technology, needing to rely upon non-HIPAA compliant platforms, taking online courses on one’s own time, and the total cutoff of those most marginalized. The PERCS framework encourages organizations to address the root causes of these barriers proactively and could be leveraged to help organizations develop pathways to address the ethical issues identified in this study.

The themes of access and digital inclusion identified in this study also evoke the public health theory of ‘intervention-generated inequalities’ (IGIs), a phenomenon in which an intervention that benefits the health outcomes of a population overall may inadvertently widen the gulf between the health outcomes of the socioeconomically advantaged and disadvantaged [23]. Several of the respondents in the current survey shared concerns about the digital divide and potential inequities. A key implication of the current study is that organizations need to proactively build and provide the infrastructure required for providers and clients to engage in telehealth sessions effectively so that the many advantages of telehealth cited in the survey can be realized equitably by all clients.

### Limitations

Results of this study should be considered in the context of several limitations. Most notably, the sample was self-selecting; only 41% of respondents to the original survey responded to the question at hand. The reasons motivating respondents’ choice to reply and the extent to which the experiences and perceptions of responders differed from non-responders are both unknown. Although the original survey asked that respondents answer on behalf of their organization, it is plausible that respondents centered their personal experiences in their responses. This tension was evident in such responses as those discussing the experience of having children at home, some of which discussed challenges facing the organization as a whole and some of which were far more personal. In addition, a convenience sample was used for the original survey, consisting of individuals who utilize MHTTC and ATTC services. The respondents from the SUD survey represented only 33 American states and Puerto Rico; those from the MH survey represented only 25 American states, the District of Columbia, Puerto Rico, and the United States Virgin Islands. Survey respondents might also have differed in their understanding of the purpose of the question (i.e., whether it was intended to improve telehealth platforms, for research purposes, etc.), which could have influenced the specific themes raised.

## Conclusions

The current study raises several directions for future research and service provision. First, many of the respondents explicitly supported a hybrid model of treatment combining virtual and in-person treatment. A hybrid treatment model has been posited as a way forward as post-COVID-19 public safety precautions subside [24, 25]. One option to apply such a model is to enable the provider and client to collaborate to choose the best treatment modality. This approach would be established based on a number of factors such as provider and client access to the technology infrastructure, client preference, provider discretion, and specific barriers impeding access to services [26].

Second, consistent with prior research [25, 27], this study highlighted organizations’ desire to continue easing regulatory restrictions on telehealth. Many respondents felt that their ability to offer virtual services hinged upon whether COVID-19-era eased reimbursement policy would continue; additionally, several respondents asserted that non-licensed professionals and other staff should be able to use telehealth. The frequency and vehemence with which respondents shared their perspectives that reimbursement regulations should continue, both in this data and in other research, demonstrates the high demand for telehealth in the behavioral healthcare system.

In terms of research implications, research comparing treatment outcomes of those receiving in-person and virtual services will be necessary to undergird organizations’ financial support, and perhaps also legislative support, for virtual SUD and MH services. Future research would also benefit from comparing the outcomes of in-person and telehealth treatment performed during the COVID-19 pandemic in particular.

## Data Availability

The datasets used and/or analyzed during the current study are available from the corresponding author on reasonable request.

## Abbreviations

ATTC: Addiction Technology Transfer Centers
COVID-19: Novel coronavirus 2019
FQHC: Federally Qualified Health Center
HIPAA: The Health Insurance Portability and Accountability Act of 1996
MH: Mental health
MHTTC: Mental Health Technology Transfer Centers
SUD: Substance use disorder

## Footnotes

1 A Federally Qualified Health Center (FQHC) is a community-based healthcare provider designated by the United States Health Resources and Services Administration (HSRA) to receive funds to provide services in underserved areas [11].
